# Modelling of Fluidised Geomaterials: The Case of the Aberfan and the Gypsum Tailings Impoundment Flowslides

**DOI:** 10.3390/ma10050562

**Published:** 2017-05-20

**Authors:** Paola Dutto, Miguel Martin Stickle, Manuel Pastor, Diego Manzanal, Angel Yague, Saeid Moussavi Tayyebi, Chuan Lin, Maria Dolores Elizalde

**Affiliations:** 1 Bundesanstalt für Materialforschung und-prüfung, Berlin 12167, Germany; 2 Department of Applied Mathematics, ETSI Caminos, Universidad Politécnica de Madrid, Profesor Aranguren s/n, 28040 Madrid, Spain; miguel.martins@upm.es (M.M.S.); manuel.pastor@upm.es (M.P.); diego.manzanal@gmail.com (D.M.); angel.yague@upm.es (A.Y.); saeid.moussavita@alumnos.upm.es (S.M.T.); 3 INTECIN-CONICET-UBA-UNPSJB, Buenos Aires 1426, Argentina; 4 College of Water Conservancy and Hydropower, Hohai University, Nanjing 210098, China; hhulinchuan@gmail.com; 5 Consejo Superior de Investigaciones Científicas, Albasanz, 26-28, 28037 Madrid, Spain; lola.elizalde@cchs.csic.es

**Keywords:** SPH, landslide propagation modelling, Perzyna viscoplasticity, Aberfan flowslide, Gypsum tailings impoundment flowslide, fluidised behaviour

## Abstract

The choice of a pure cohesive or a pure frictional viscoplastic model to represent the rheological behaviour of a flowslide is of paramount importance in order to obtain accurate results for real cases. The principal goal of the present work is to clarify the influence of the type of viscous model—pure cohesive versus pure frictional—with the numerical reproduction of two different real flowslides that occurred in 1966: the Aberfan flowslide and the Gypsum tailings impoundment flowslide. In the present work, a depth-integrated model based on the $v-p_{w}$ Biot–Zienkiewicz formulation, enhanced with a diffusion-like equation to account for the pore pressure evolution within the soil mass, is applied to both 1966 cases. For the Aberfan flowslide, a frictional viscous model based on Perzyna viscoplasticity is considered, while a pure cohesive viscous model (Bingham model) is considered for the case of the Gypsum flowslide. The numerical approach followed is the SPH method, which has been enriched by adding a 1D finite difference grid to each SPH node in order to improve the description of the pore water evolution in the propagating mixture. The results obtained by the performed simulations are in agreement with the documentation obtained through the UK National Archive (Aberfan flowslide) and the International Commission of large Dams (Gypsum flowslide).

## 1. Introduction

Flowslides are rapid flows, either saturated or unsaturated, where the material has a high compaction tendency, a low density and is characterised by a metastable structure. Since flow failures experience a sudden loss of strength followed by a very rapid development of deformations, their effects are often much more dramatic and devastating than other types of landslides. Thus, the prediction of flowslides’ propagation distances, velocity and pore water pressure will be of great human and economic benefit and an effective way of identifying and designing appropriate preventive flowslide measures.

In order to make such a prediction, fundamentals are needed: (i) a coupled mathematical model, (ii) a suitable rheological model for the considered material and (iii) a numerical model where (i) and (ii) are implemented.

Moreover, depth-integrated models have been found to provide a suitable approximation for many 3D problems. These types of models result in an excellent compromise between accuracy and computational cost and have been applied to landslides since the work of Savage and Hutter [1]. Other authors have followed the same technique, such as the work of Iverson and Denlinger [2], Hutter and Koch [3], Naaim et al. [4], Laigle and Coussot [5], Pitman and Le [6], McDougall and Hungr [7], Rodriguez-Paz and Bonet [8], Mangeney-Castelnau [9], Lajeunesse et al. [10] and Pastor et al. [11,12,13].

Pore pressures play a paramount role in the behaviour of soil structure and, indeed, their variation may induce failure, but in most of the mentioned models it is not taken into account. Hutchinson [14], Iverson and Denlinger [2], Pastor et al. [15] and Quecedo et al. [16] are among the authors who provided information on the pore water pressure developing inside the landslide. In the case of depth-integrated models, all information concerning vertical profiles was condensed on a single variable describing basal pore pressure, and its evolution was modelled using simplified approaches. 

The mathematical model considered in the present work combines a depth-integrated description of the coupling soil skeleton and pore fluid with a 1D model dealing with pore pressure evolution within the soil mass at each computational step of the flowslide’s propagation. In this way, pore pressure changes caused by different phenomena such as changes in height, changes of basal surface permeability or changes in total stress may be taken into account. 

The consistent study of both the triggering and propagation phases presents the problem of having to use a constitutive model for the first part of the analysis and a rheological model for propagation. Among the most used constitutive models, it is worth mentioning the work of Desai and Siriwardane [17], Cambou and Di Prisco [18], Kolymbas [19] or Zienkiewicz et al. [20]. Concerning the rheological models, they have to be able to reproduce the behaviour of fluidised materials and they have to include mechanisms for the changes in pore pressure.

In the case of cohesive fluids, exhibiting a yield stress, it is worth mentioning the work of Hohenemser and Prager [21], Oldroyd and Wilson [22], Coussot and Piau [23], Coussot [24,25] Dent and Lang [26] and Locat and Demers [27]. 

In this paper, an alternative model based on classical rheological models and on Perzyna viscoplasticity, thinking of viscoplasticity as the key to close the gap between the triggering and the propagation mechanism, will be used (Pastor et al. [28]).

The Smoothed Particle Hydrodynamics (SPH), a Lagrangian meshless numerical technique, has been chosen to discretise the depth-integrated equations of the mathematical model; the main advantage, regarding other well-known techniques such as the Finite Element Method or the Discrete Element Method, being the computational cost. 

The goal of this study is to apply the SPH depth-integrated numerical model, which includes a sub-model able to predict the evolution of the pore water pressure, to gain insight and reproduce the propagation phase of two man-made hazards that occurred in 1966: the Gypsum tailings impoundment and the Aberfan flowslides. 

This paper is structured as follows: for the sake of completeness, in section two, the depth-integrated mathematical model based on $v-p_{w}$ Biot–Zienkiewicz formulation, including a description of the evolution of the pore pressures within the propagating mixture, is described. In section three, a brief description of the rheological models for fluidised soils, including a description of pure cohesive and frictional viscous models, is established. In section four, some details of the numerical resolution of the equations are set. In section five, the described numerical method is applied to the Aberfan and the Gypsum tailings impoundment flowslides. Some conclusions are established in section six.

## 2. Mathematical Model

### 2.1. Solid Skeleton Pore Fluid Coupled Mathematical Model 

The starting point for the mathematical model considered in the present work, in order to describe the behaviour of the mixture that propagates within the landslide, is the well known $v-p_{w}$ Biot-Zienkiewicz model [28] which is composed of the balance momentum of the mixture (1) and the combined pore fluid balance of mass and linear momentum (2)

(1) $$\rho \frac{dv}{dt}=\rho b+div\;\sigma$$

(2) $$div\left(-k_{w}grad\;p_{w}\right)+div\;v=0$$

Here v is the velocity of the solid skeleton, ρ is the mixture density, σ is the total Cauchy stress tensor while b is the gravity acceleration vector. The symbol $\frac{d}{dt}$ in (1) is the material time derivative with respect to the solid skeleton. It is defined by $\frac{dv}{dt}=\frac{\partial v}{\partial t}+grad\;v\cdot v$ where *grad* means gradient operator with respect to the spatial position. In both equations, the operator *div* is the divergence that in (2) is associated with *grad*. Moreover, in (2), $p_{w}$ is the pore fluid pressure and $k_{w}$ is the permeability coefficient. Incompressibility of pore fluid and solid skeleton particles has already been assumed in (2).

Most of the landslides are shallow, i.e., if *L* is a characteristic length of the landslide and H is a characteristic depth of the sliding mass, then ε=H/L≪1. This geometric characteristic suggests, after a dimensionless analysis is performed, that the decomposition of the velocity field v [29] in a vertical consolidation component $v^{c}$ and a propagation component $v^{p}$ is possible and it is done as follows

(3) $$v=v^{c}+v^{p}$$

allowing the description of the landslide evolution as the combination of two different physical phenomena, namely, pore pressure evolution and propagation. Therefore, the mixture initially describe by (1) and (2), can be expressed by the following set of equations

(4) $$\begin{array}{l} \rho \frac{dv^{p}}{dt}=\rho b+div\;\sigma \\ div\;v^{p}=0 \end{array}$$

(5) $$div\;v^{c}=k_{w}\frac{\partial^{2}p_{w}}{\partial x_{3}^{2}}$$

where (4) governs the propagation of the mixture as an incompressible viscous fluid while (5) governs the evolution of the pore water pressure through a vertical consolidation process. The complete set of governing Equations (4) and (5) can be applied with confidence to the case of flowslides, where the mixture presents a medium permeability and consolidation while propagation phenomena are developed with a similar order of magnitude in time.

The principal component of volume changes within a spreading mixture governed by the set of Equations (4) and (5) is due to a vertical consolidation process. This behaviour, specific to flowslides, might not be applied to debris flows, where very high permeabilities may be observed and other sources of volume changes should be considered [30]. For the case of mudflows, where very low permeabilities are observed and the time required to develop consolidation is much larger than the time required for the propagation of the mixture, incompressible behaviour is usually observed. Therefore, if a mudflow is to be modelled, Equation (5) can be neglected keeping (4) as the main set of governing equations.

**Figure 1:**
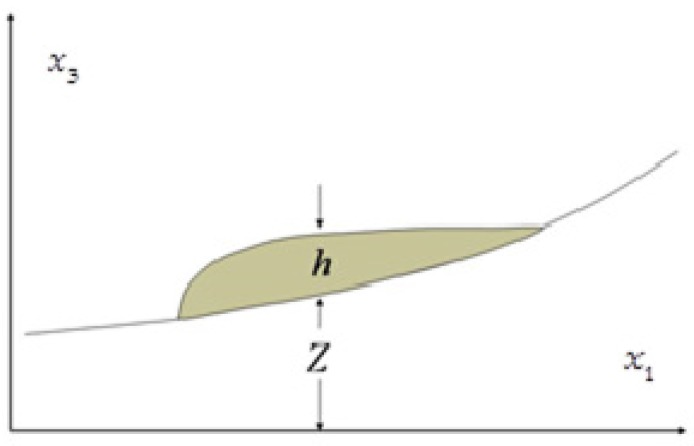
Reference system and notation used in the analysis [28].

In order to be able to solve the Equations (4) and (5), appropriate initial and boundary conditions are needed. Regarding the boundary conditions (Figure 1), two different types of boundaries are considered: a no-slip condition at basal surface, defined by $x_{3}=Z\left(x_{1},x_{2},t\right)$, and a free surface, defined by $x_{3}=h\left(x_{1},x_{2},t\right)+Z\left(x_{1},x_{2},t\right)$.

As per the previous definition, the basal surface Z varies with time. Under this assumption, erosion can be considered in the mathematical description of the landslide by defining the erosion rate $e_{R}$ to be

(6) $$e_{R}=-\frac{\partial Z}{\partial t}$$

The system of partial differential Equations (4) and (5) are solved in the present work by numerical techniques. But first, in the following sections, system (4) is integrated into depth while Equation (5) is transformed into a diffusion-like equation.

### 2.2. Depth-Integrated Mathematical Model

In what follows, superindex p in (4) is dropped for brevity. Resolution of the above defined 3D boundary value problem is a formidable task if no reasonable simplifications are considered. In this context, depth-integrated models are a convenient simplification of 3D models, providing an acceptable compromise between computational cost and accuracy [30].

In order to obtain the depth integrated version of the system (4), the quasi-material derivative $\frac{\bar{d}}{dt}$ is first introduced by the expression

(7) $$\frac{\bar{d}}{dt}=\frac{\partial }{\partial t}+{\bar{v}}_{j}\frac{\partial }{\partial x_{j}},\;j=1,2$$

where ${\bar{v}}_{j}$ is the j component of the average value of the velocity v over the flow depth h defined by

(8) $$\bar{v}=\frac{1}{h}\int_{Z}^{Z+h}v\;dx_{3}$$

Integrating into depth, taking into account Leibniz’s rule for differentiating integrals and considering the boundary conditions at the basal and free surface, the depth-integrated balance of mass reads as

(9) $$\frac{\bar{d}h}{dt}+hdiv\;\bar{v}=e_{R}$$

where $e_{R}$ is the erosion rate defined by (6), while $div\;\bar{v}=\frac{\partial {\bar{v}}_{1}}{\partial x_{1}}+\frac{\partial {\bar{v}}_{2}}{\partial x_{2}}$. 

If a depth integration approach is applied to the balance of linear momentum and if an internal viscosity force within the incompressible mixture is neglected, as compared with the viscous resistance opposed by the basal surface to the displacement of the mass, the following expression is obtained [30]

(10) $$\rho h\frac{\bar{d}\bar{v}}{dt}=-\frac{1}{2}\rho \;grad(gh^{2})-\rho gh\;grad(Z)+\tau_{b}-\rho \;e_{R}\bar{v}$$

where g is the acceleration of gravity, ρ is the mixture density, h is the depth of the flow, v is the average velocity over h, Z is the basal surface, $e_{R}$ is the erosion rate and $\tau_{b}$ is the basal shear stress that depends on the rheological law considered. Also, in (10), $grad=\left(\frac{\partial }{\partial x_{1}},\frac{\partial }{\partial x_{2}}\right)$.

### 2.3. Pore Pressure Evolution in Landslides

In what follows, superindex c in (5) is dropped for brevity. Bearing in mind the relation $div\;v=\frac{d\varepsilon_{v}}{dt}$, where $\varepsilon_{v}$ is the volumetric deformation, it is assumed that the time rate of change of the volumetric deformation $\varepsilon_{v}$ can be related to the time rate of variation of effective confining pressure $p^{\prime }$ by

(11) $$\frac{d\varepsilon_{v}}{dt}=-\frac{1}{K_{v}}\frac{dp^{\prime }}{dt}$$

where $K_{v}$ is a suitable stiffness modulus, $p^{\prime }=-\frac{1}{3}tr\;\sigma^{\prime }$, and $\sigma^{\prime }$ is the effective stress tensor.

If the skeleton is elastic, $K_{v}$ is the elastic volumetric stiffness ratio. For example, for oedometric conditions, the volumetric stiffness ratio is considered as the oedometric modulus, $K_{v}=E_{m}$. Taking into account that $p^{\prime }=p+p_{w}$, Equation (5) can be rewritten as

(12) $$\frac{dp_{w}}{dt}=-\frac{dp}{dt}-K_{v}k_{w}\frac{\partial^{2}p_{w}}{\partial x_{3}^{2}}$$

**Figure 2:**
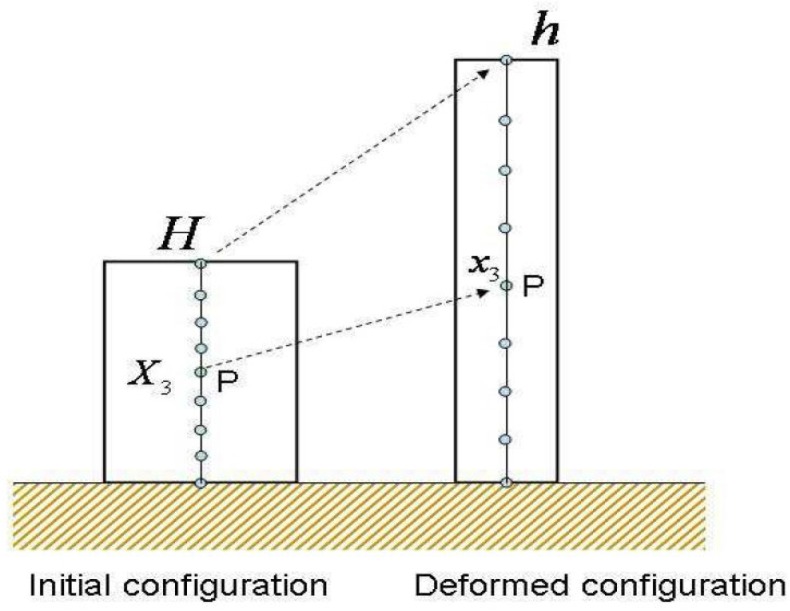
Deformation of a soil column [28].

In order to solve this equation, the landslide mass will be decomposed into differential elements of volume having a height *h* and a differential cross section dA at each time *t*, as shown in Figure 2. 

The changes of the total mean confining pressure p are mainly caused by the height variation. So, for the differential volume in Figure 2, the total stress will vary as follows:

(13) $$\sigma_{3}=-\rho (h-x_{3})g$$

Then, the total stress $\sigma_{3}$, which depends on h, varies with it as

(14) $$\frac{d\sigma_{3}}{dt}=-\rho g\left(\frac{dh}{dt}-\frac{dx_{3}}{dt}\right)=-\rho g\frac{dh}{dt}\left(1-\frac{x_{3}}{h}\right)$$

Concerning the effective stress, it is observed that

(15) $$\frac{d{\sigma^{\prime }}_{3}}{dt}=-\rho g\frac{dh}{dt}\left(1-\frac{x_{3}}{h}\right)+\frac{dp_{w}}{dt}$$

and by considering the relation $dp^{\prime }=\alpha d{\sigma^{\prime }}_{3}$, it is found that

(16) $$\frac{dp_{w}}{dt}=\rho g\frac{dh}{dt}\left(1-\frac{x_{3}}{h}\right)+\frac{K_{v}}{\alpha }k_{w}\frac{\partial^{2}p_{w}}{\partial x_{3}^{2}}$$

which is the equation describing the evolution of the pore pressure along $x_{3}$. Equation (16) has to be complemented with an initial and boundary condition at $x_{3}=Z$ and $x_{3}=Z+h$. For example, it can be considered zero at the surface and zero flow at the bottom. 

Summarising, the mathematical model considered in the present work to reproduce the principal features of a landslide consists of:

1. Depth-integrated equations
  (17) $$\frac{\bar{d}h}{dt}+hdiv\;\bar{v}=e_{R}$$
  (18) $$\rho h\frac{\bar{d}\bar{v}}{dt}=-\frac{1}{2}\rho \;grad(gh^{2})-\rho gh\;grad(Z)+\tau_{b}-\rho \;e_{R}\bar{v}$$
2. A pore pressure evolution equation
  (19) $$\frac{dp_{w}}{dt}=\rho g\frac{dh}{dt}\left(1-\frac{x_{3}}{h}\right)+\frac{K_{v}}{\alpha }k_{w}\frac{\partial^{2}p_{w}}{\partial x_{3}^{2}}$$

Before performing the numerical approach, a rheological model should be established in order to clarify the expression for the basal shear stress $\tau_{b}$ and its possible relation with the basal excess pore pressure. This will be clarified in the following section.

## 3. Rheological Models for Fluidised Soils

### 3.1. Introduction

In this section, the behaviour of fluidised geomaterials in fast landslides will be discussed. Once failure has been triggered, the behaviour of the soil mass is closer to that of fluids than to solids. This is why rheological models are used to describe the behaviour of such hazards. There are many types of materials involved in fast landslides, from assemblies of rock blocks to mixtures of clay and water. 

Because of the computational cost of full 3D models, researchers have favoured the use of simpler depth-integrated models, as described previously, where the flow structure is lost and the basal friction is obtained from the depth average velocity.

The purpose of this section is then to describe three rheological models, two of them widely used and a new one based on Perzyna viscoplasticity for the study of landslides’ propagation. The scope is to understand how these models provide an expression of basal friction using the hypothesis of simple shear Infinite Landslide Model, once the depth average velocity is known.

### 3.2. Pure Cohesive Viscoplastic Fluid: Bingham Model

The Bingham model includes two material parameters, the yield stress below which the material does not flow, and the viscosity. It was introduced by Bingham [31] in 1922. The expression for the Bingham model (where $\tau_{y}$ is the yield stress) is written as:

(20) $$\tau =\tau_{y}+\mu \left(\frac{\partial v_{1}}{\partial x_{3}}\right)$$

Depending on the fluid phase viscosity, mudflows, lahars and debris flow can be modelled as viscoplastic fluids with Bingham-like models. Considering a Bingham fluid initially at rest and increasing the shear stress, the fluid will start moving only when the shear stress reaches $\tau_{y}$. This behaviour creates what is generally called a “plug” or a zone where the velocity is constant and the rate of deformation is zero. 

Concerning the bottom friction, it is assumed that it can be approximated under the hypothesis of simple shear flow conditions. As described in [15], the shear stress at the bottom $\tau_{b}$ can be related to the depth-averaged velocity with the following expression:

(21) $$\bar{v}=\frac{\tau_{b}h}{6\mu }{\left(1-\frac{\tau_{y}}{\tau_{b}}\right)}^{2}\left(2+\frac{\tau_{y}}{\tau_{b}}\right)$$

### 3.3. Pure Frictional Viscoplastic Fluid

Frictional viscoplastic fluids are used to model fast landslides where friction is important. If the cohesion is assumed to be zero and using

(22) $$\sigma_{13}=\sigma_{31}=s+\mu {\left(\frac{\partial v_{1}}{\partial x_{3}}\right)}^{m}$$

it is easy to obtain

(23) $$\tau (z)-s(z)=\mu {\left(\frac{\partial v_{1}}{\partial x_{3}}\right)}^{m}$$

where $\tau (z)=\tau_{b}\left(1-\frac{z}{h}\right)$. *h* is the total height, $\tau_{b}=\rho gh\sin\theta$ is the basal shear stress with density ρ and $s(z)=-\rho_{d}^{'}g(h-z)\cos\theta \tan\varphi$ is the strength along *z*, being $\rho_{d}^{'}=\rho -\rho_{w}$. The velocity profile can then be obtained as

(24) $$v=v_{h}\left\{1-{\left(1-\frac{z}{h}\right)}^{\frac{1+m}{m}}\right\}$$

and depth integrating

(25) $$\bar{v}=v_{h}\left(\frac{1+m}{1+2m}\right)$$

where $v_{h}$ is the velocity at the surface.

The basal shear stress becomes then

(26) $$\tau_{b}=s_{b}+{\left(\frac{1+2m}{m}\right)}^{m}\frac{1}{h^{m}}\mu {\bar{v}}^{m}$$

where $s_{b}$ is the shear strength at the bottom.

### 3.4. Perzyna-Based Rheological Model for Frictional Materials

Viscoplastic models were found to provide a suitable and more economic approach than classical plasticity models when computing failure loads and mechanisms [32]. In the case of soils, viscoplastic models have been applied both to cohesive [33,34,35] and frictional materials. They have been found to reproduce well slow landslide movements [32,35,36].

There exists an interesting similitude between the viscoplastic fluid rheological model of the type $\tau =s+\mu {\left(\frac{\partial v}{\partial z}\right)}^{m}$, from where the shear strain can be written as

(27) $$\left(\frac{\partial v}{\partial z}\right)=\frac{1}{\mu^{\frac{1}{m}}}{\left(\tau -s\right)}^{\frac{1}{m}}$$

and the Perzyna elasto-viscoplastic models. In the latter models, the relation between the effective stress and the rate of deformation tensor is given by

(28) $$\sigma '=D^{e}:(d-d^{vp})$$

Above, $D^{e}$ is the elastic constitutive tensor, d is the rate of deformation tensor and $d^{vp}$ is the viscoplastic component. The viscoplastic component of the rate of the deformation tensor is given by Perzyna [37,38] as

(29) $$d^{vp}=\gamma n_{g}\langle \Phi (F)\rangle$$

where 〈〉 represents the Macaulay brackets and γ is the fluidity parameters. $n_{g}$ is a unit norm tensor characterising the direction of the plastic flow and Φ(F) is an arbitrary function.

The function chosen here has the following form:

(30) $$\Phi (F)={\left(\frac{F-F_{0}}{F_{0}}\right)}^{N}$$

where *N* is a model parameter and *F* a function describing a convex surface in the stress space. The value $F_{0}$ corresponds to the value of stress below which no viscoplastic flow occurs. If *F* is chosen to be equal to τ and $F_{0}$ is chosen to be the cohesive-frictional strength *s*, then the rate of viscoplastic strain can be rewritten as

(31) $$\frac{\partial v}{\partial z}=\gamma {\left(\frac{\tau -s}{s}\right)}^{N}$$

where γ=1/μ1m, N=1m and the elastic contributions are neglected.

Assuming a simple shear Infinite Landslide Model where τ=ρg(h−z)sinθ and s=ρg(h−z)cosθtanφ, then Equation (31) becomes

(32) $$\frac{\partial v}{\partial z}=\gamma {\left(\frac{\tan\theta -\tan\varphi }{\tan\theta }\right)}^{N}=Const$$

which results in a linear velocity profile.

A new simple rheological law based on Perzyna viscoplasticity for frictional materials is then easy to find, being the expression of the basal friction $\tau_{b}$

(33) $$\tau_{b}=s_{b}\left[{\left(\frac{\bar{v}2\mu }{h}\right)}^{\frac{1}{N}}+1\right]$$

where $s_{b}=\sigma_{3b}\tan\varphi$ is the basal shear strength (z=0), μ is the viscosity [s] and $\bar{v}$ is the depth-integrated velocity. More details on the model can be found in [28,29,30]. 

## 4. Depth-Integrated SPH Model Coupled with a Finite Difference Scheme for Pore Pressure

The Smoothed Particle Hydrodynamics (SPH) is a meshless method, which has been applied to a large variety of problems, introduced independently by Lucy [39] and Gingold and Monaghan [40]. The SPH is a numerical technique able to simulate the propagation of fast landslides, which are treated as fluidised masses. It is based on the approximation of given properties and their spatial derivatives by integral approximation defined in terms of smoothed functions or kernel functions. An interpolation process calculates the relevant properties of each “particle” over neighbouring “particles”. Therefore, the SPH is based on introducing a set of nodes together with a set of nodal variables. For landslides’ problems, these variables are the height of the landslide at node I, the depth-averaged 2D velocity, the surface force vector at the bottom and the pore pressure at the basal surface. Details of the formulation can be found in [41].

In the present work, the previous development of the GEOFLOW-SPH code [11] has been enriched adding a 1D finite difference grid to each SPH node, in order to improve the description of the pore water evolution in the propagating mixture. The Finite Difference (FD) scheme chosen is explicit and is centred in space and forward in time (FCTS). At every node and time step, critical step times of FD and SPH are compared, and, if necessary, the pore pressure equation is solved using internal sub-steps.

## 5. Application

### 5.1. Introduction

In this section, a depth-integrated model based on the $v-p_{w}$ Biot–Zienkiewicz formulation, enhanced with a diffusion-like equation to account for the pore pressure evolution within the soil mass, is applied to the Aberfan flowslide and Gypsum tailings impoundment flowslide that both occurred in 1966. In the case of the Gypsum flowslide, a pure cohesive viscous model—the Bingham model—is considered, while for the Aberfan flowslide, a frictional viscous model based on Perzyna viscoplasticity is developed.

### 5.2. East Texas Gypsum Tailings Failure (1966)

Tailings impoundments involve very loose materials. Failure of the dam results in the propagation of the fluidised materials, which behave like cohesive-viscous fluids. A representative case for which there is available information is that of East Texas Gypsum tailings impoundment, which failed in 1966. It has been described by Jeyapalan et al. [42], and Pastor et al. [12], who modelled the problem using a depth-integrated finite element model assuming that the material behaved as a Bingham fluid.

The purpose of this section is to show how the problem can be modelled using a depth-integrated SPH model.

According to the description provided in [42], the impoundment was rectangular; the tailings having reached a depth of 11 m at the time the failure took place. The failure affected a length of the dyke of 140 m. The material propagated some 300 m beyond the dyke before stopping. The average velocity was in the range of 2.5–5 m/s, with a propagation time close to 60–120 s.

This failure released an amount of approximately 100,000 m^3^ of tailings (80,000–130,000 m^3^ according to Jeyapalan et al. [42]).

One key point is the rheological model and its parameters. Here, we have used a Bingham model, with a yield stress of 750 Pa and a viscosity of 35 Pa·s, obtained by back analysis. The tailings were non-plastic silts, according to Jeyapalan, with D50 of 0.07 mm, a density of the particles of 2450 kg/m^3^ and a density of the mixture of 1400 kg/m^3^. 

**Figure 3:**
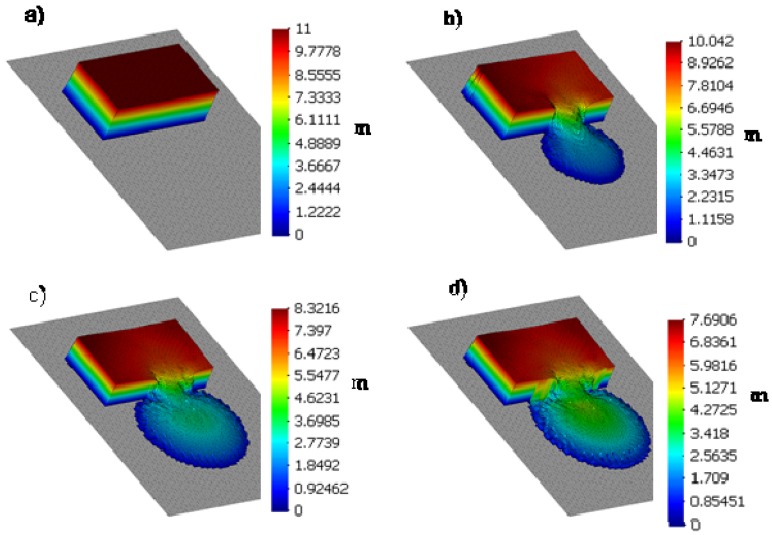
Soil height results of the analysis propagation of the tailings at a series of time stations. (**a**) *t* = 0 s; (**b**) *t* = 30 s; (**c**) *t* = 60 s and (**d**) *t* = 80 s.

**Figure 4:**
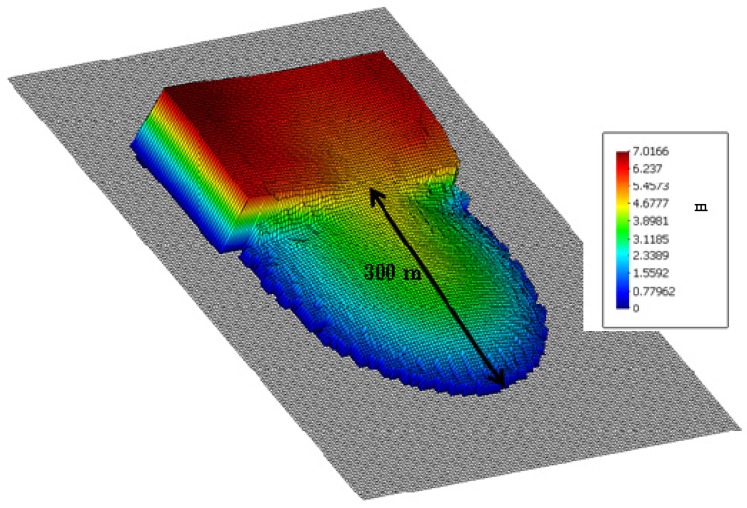
Soil height results of the analysis propagation of the tailings at *t* = 120 s with a final run out of 300 m.

We provide the height of the soil results (in meters) of the analysis in Figure 3 and Figure 4, where we have depicted the propagation of the tailings at a series of time stations (*t* = 0, 30, 60, 80 and 120 s). The results agree well with the observations, the runout onto the plane being approximately 300 m and the movement being close to zero at time 60 s. The vertical scale has been enlarged by a factor of 10. 

In this SPH simulation, we have observed that the failure propagated inside the impoundment for a distance longer than the 110 m provided in the bibliography.

Regarding the computation, we have used normalisation of the tailings height close to the dykes, in order to avoid the particle deficiency problem found close to boundaries. We have used 2485 SPH nodes in the analysis. 

### 5.3. The Aberfan Flowslide

Here, the event of the Aberfan flowslide will be analysed and the results of the simulations obtained using the mathematical and constitutive model described before will be shown. Moreover, a sensitivity analysis of the main parameters of the model is made in order to show how drastically the results change by changing the parameters model. 

Aberfan is today a former coal mining village in South Wales (UK). In 1966, a flowslide of coal waste occurred, propagating onto the village itself and provoking 144 fatalities. Information about the failure mechanism and material properties have been provided by Bishop [43,44] and Hutchinson [14]. Other raw material is also available at the UK National Archive.

The Aberfan colliery waste was tipped on the side of a hill (Tip 7) facing the village. The triggering mechanisms of the flowslide lay in the hydrogeology of the site. Due to heavy rain, in fact, artesian pore pressure rose up in the sandstone beneath the less permeable glacial deposit at the toe of the slope, causing the liquefaction of the loose waste material dumped.

Tip 7 was about 67 m in height from the toe of the slope at the moment the slide occurred on 21 October and the underlying terrain had a slope of 12 degrees. The slide moved for 275 m before dividing itself into two lobes. The larger south lobe travelled for a distance of 500 m before impacting Aberfan buildings and stopped 100 m after, for a total propagation length of 600 m with estimated velocities in the range of $4.5-9\frac{m}{s}$.

**Figure 5:**
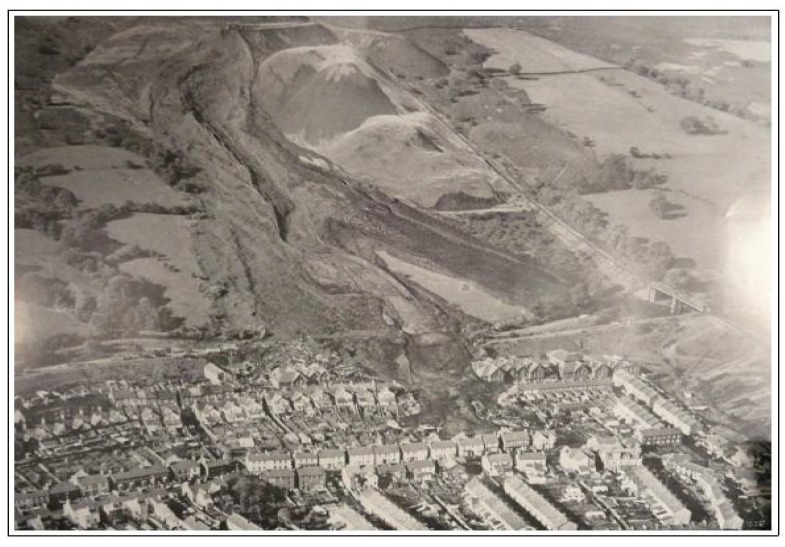
Photograph of the Aberfan disaster (from UK National Archive).

Table 1 summarises the characteristics of the flowslide while in Figure 5 it is possible to see a photograph of the disaster.

So far, only simple 1D simulations of the Aberfan flowslide have been made as shown in the work of Pastor et al. [11,30]. In this paper, the authors want to present the 3D depth-integrated model of Aberfan flowslide, showing that the patterns observed are reproduced.

**Figure 6:**
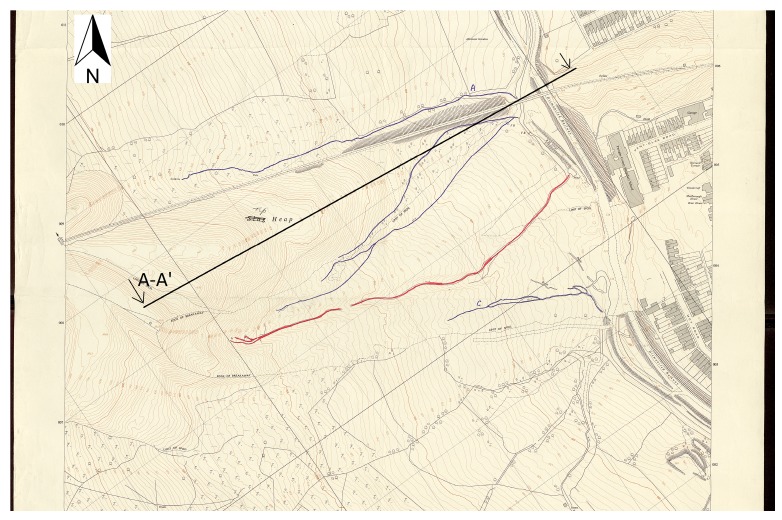
Map of Aberfan after the 1966 flowslide disaster (from UK National Archive).

**Figure 7:**
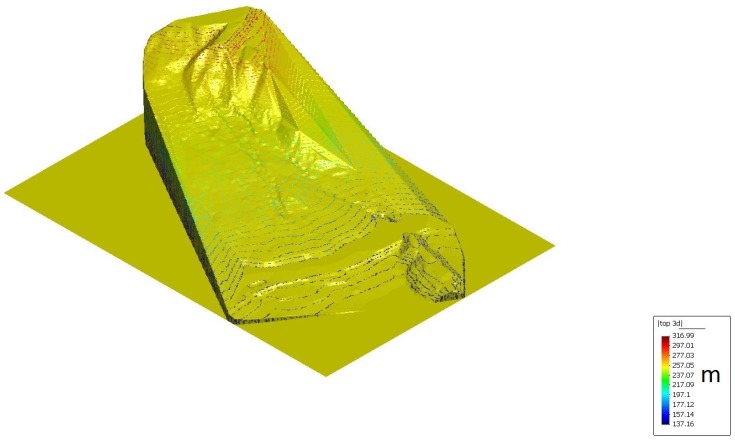
The Aberfan 3D topographic mesh generated using the map of Figure 6 as a starting point. Different colors represent different isolines’ height.

In order to do so, a proper 3D topographic mesh and an SPH mesh representing the initial mass is needed. The authors have built both of them by using topographic maps which are possible to consult in the UK National Archive. Figure 6 shows the topographic map that has been used in order to create the 3D topographical mesh (Figure 7).

The input for the topographic mesh is a Digital Terrain model with a Finite Element format with a total of 8733 nodes.

**Figure 8:**
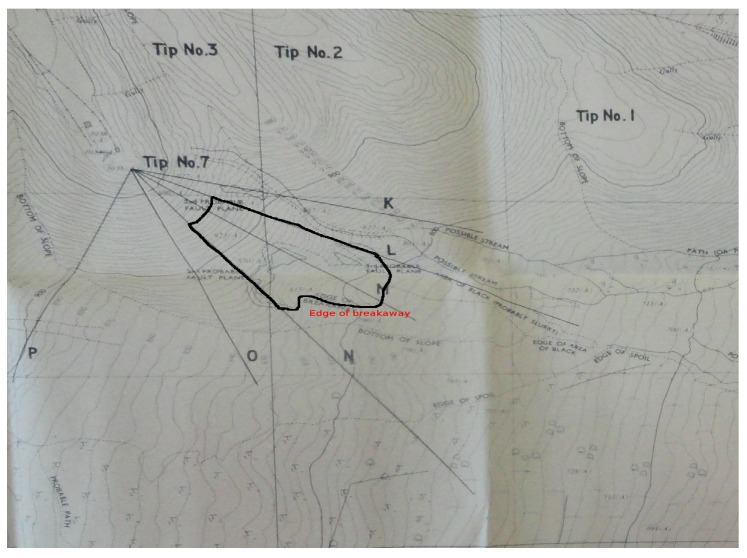
The edge of breakaway defined on Tip No. 7 (map from UK National Archive, Kew).

**Figure 9:**
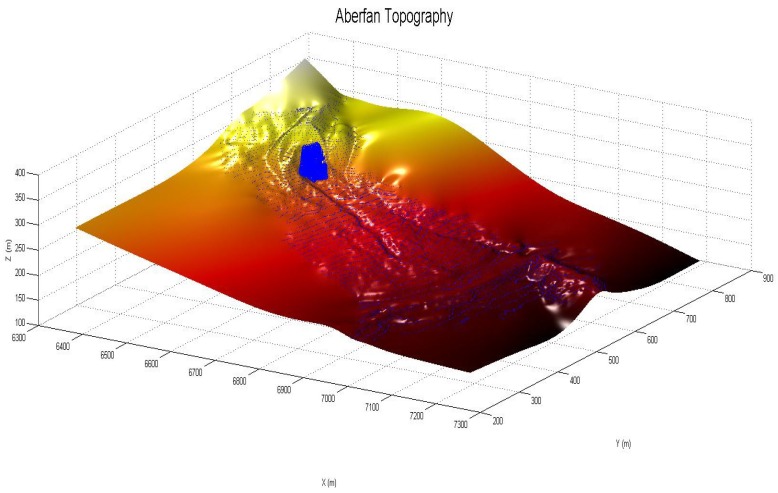
Aberfan topography and SPH nodes at the beginning of the simulation.

After identifying the edge of breakaway (Figure 8) and its correspondent height of the sliding portion of Tip 7 that generated the flowslide, the SPH mesh has been properly created, as shown in Figure 9, with 1761 nodes with an average spacing of 3 m. It has been found that an average of 1700 nodes with a spacing no larger than 4 m, reproduces well the particular phenomena.

In Table 2, the parameters used to model the Aberfan flowslide which give the best agreement with field observations are presented. Erosion has been taken into account through the erosion coefficient of the Hungr erosion law [45]. In fact, with a careful reading of the report written immediately after the disaster and available from the UK National Archives, it is possible to see that erosion is widely mentioned by the author [43]. Moreover, $p_{w}^{rel}$ represents the initial pore water pressure at the basal surface, varying between 0 and 1; 1 corresponding to liquefaction. Finally, the relative height of the basal saturated layer $h_{w}^{rel}$ was assumed to be 0.4 times the total height of the flowslide at the beginning.

### 5.4. Results, Parametric Study and Discussion

#### 5.4.1. Introduction

In this section, the results obtained with the setup described in the previous sections and the model parameters of Table 2 will be shown. Moreover, in order to prove the validity of the model itself and to justify the used parameters, a parametric study is conducted. This parametric study has the objective of showing the non-negligible differences that arise when it comes to choosing the right parameters which allow the description of the phenomena. In particular, the results shown and considered of most importance by the authors are:

- the height of propagation
- the propagation profile

In order to do so, the parameters that will vary in the simulations will be the angle of friction φ and viscosity factor μ, specific to the Perzyna-based model, and the erosion rate of growth.

#### 5.4.2. Simulation Results

**Figure 10:**
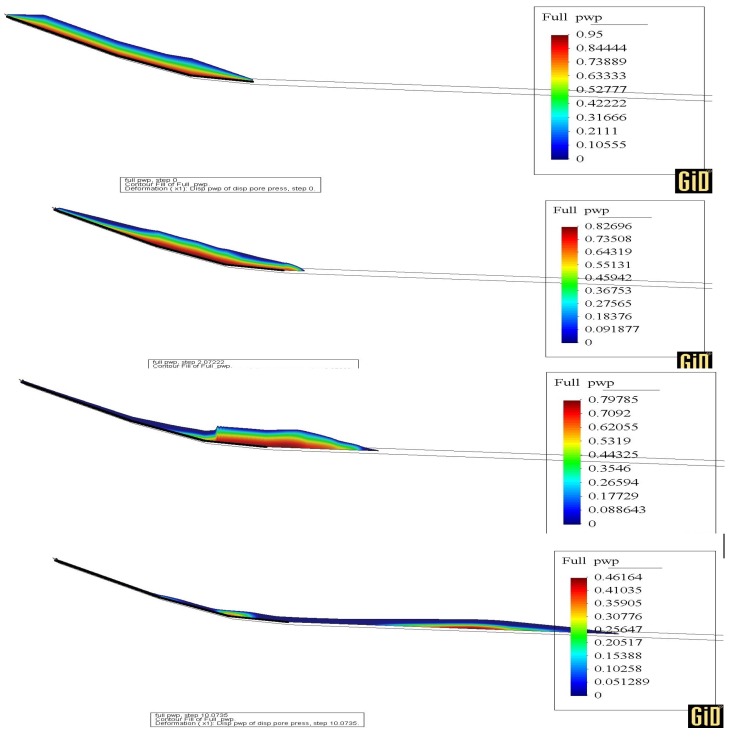
Pore water pressure contour at 0, 2, 5 and 10 s.

In Figure 10, the pore pressure contours evolution is presented at 0, 2, 5 and 10 s. Please note that in order to improve readability, the saturated layer has been expanded and now it occupies the whole mass. This is possible because we are considering the depth of the basal saturated layer proportional to the one of the landslide.

**Figure 11:**
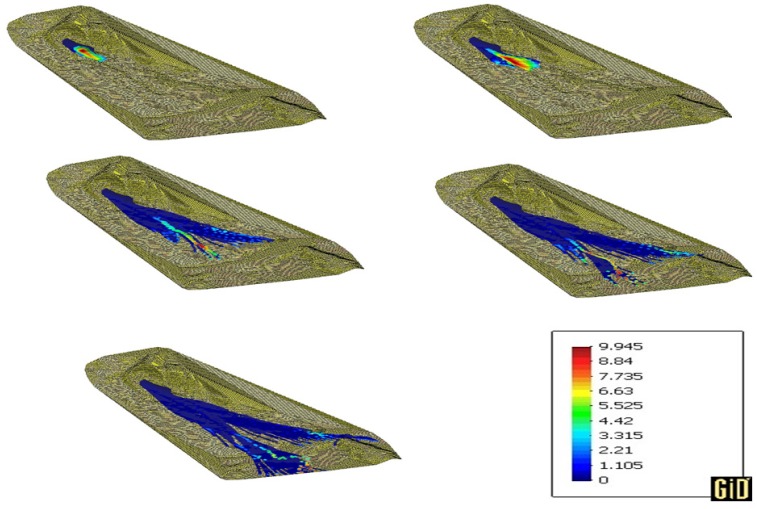
Propagation phase of the Aberfan flowslide with the parameters of Table 2. Colors refer to the height of the soil.

The results of the propagation and height of the soil obtained with the parameters described in Table 2 are shown in Figure 11 at time 5, 10, 25, 35 and 50 s. Results satisfactorily reproduce the flowslide. The legend in the picture refers to the height of the soil in meters at 50 s. It is possible to note that the final height of the soil of the left lobe at 50 s is almost 10 m which matches with the real height reported in [43]. Furthermore, the SPH program reproduces well the division of the flowslide into two lobes. Results of the soil height also match well with the one-dimensional results obtained by Pastor et al. [11,30]. 

#### 5.4.3. Parametric Study

After showing the results of the simulation, a parametric study is now shown in order to understand the difference that other sets of parameters might have on the simulation results. Table 3 shows the parameters chosen for every simulation for a total of six simulations to compare, including the original one displayed above.

**Figure 12:**
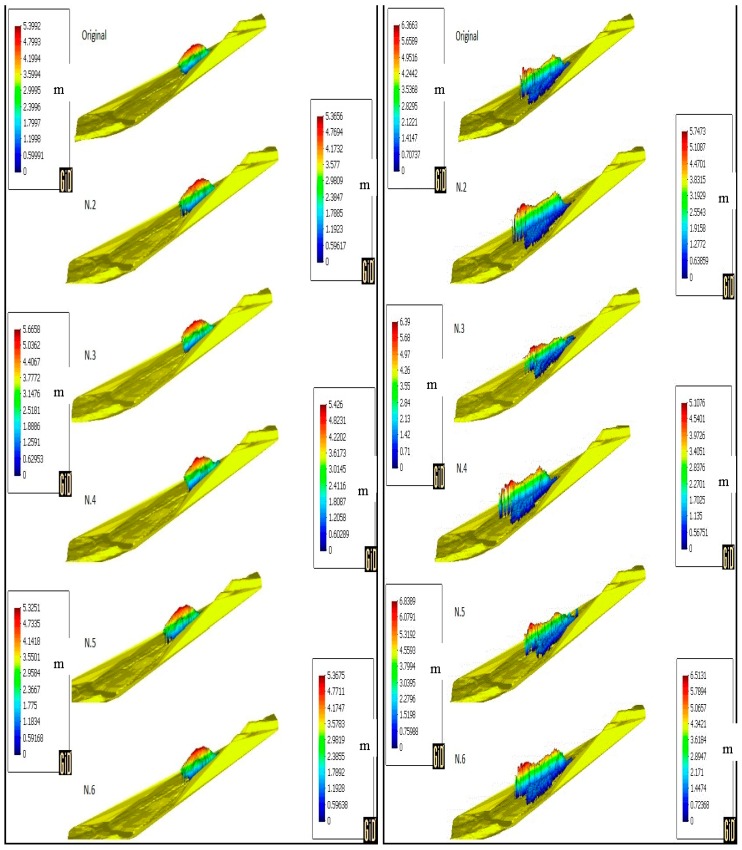
Height profile of the sliding mass at 10 s (**left**) and 20 s (**right**) for the simulations with the parameters in Table 3.

**Figure 13:**
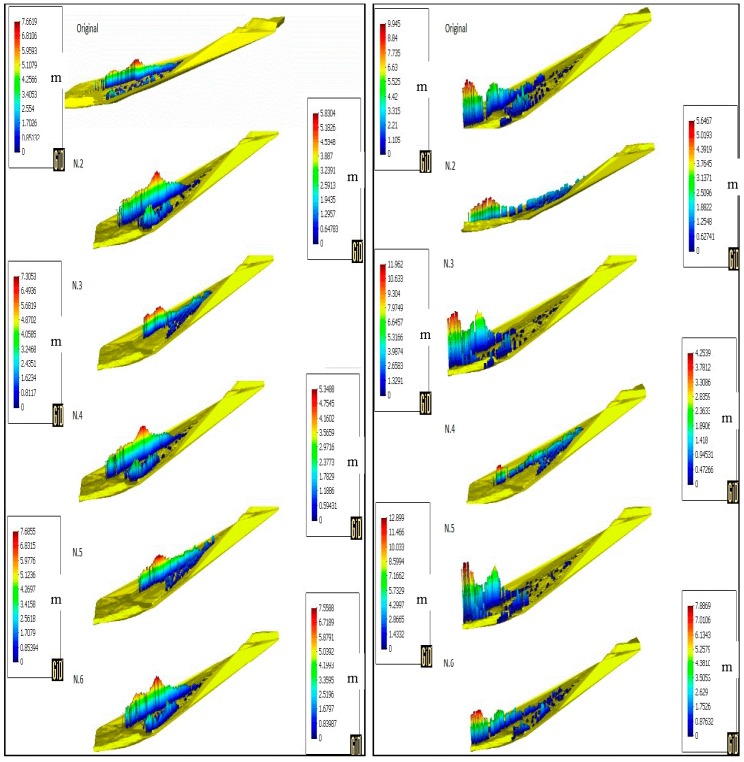
Height profile of the sliding mass at 30 s (**left**) and 50 s (**right**) for the simulations with the parameters in Table 3.

At first, the difference in the soil height is shown. Profiles are displayed at 10 s, 20 s, 30 s and 50 s in Figure 12 and Figure 13. In Figure 6, line A-A’ shows the profiles’ perspective. In order to improve the results’ readability, Table 4 summarises the values of the maximum height reached at every stage.

The height of the flowslide at the end of its propagation was observed to be 10 m when hitting the school building of the village [43]. It can be observed that by changing the parameters of the model (Table 3), the final height varies substantially. In the case of a higher viscosity value (N.2), zero erosion (N.4) or higher friction angle (N.6), the maximum height at the end of the simulation is far lower than what it is supposed to be. On the other hand, by lowering the viscosity value (N.3) or lowering the friction angle (N.5), the height reached exceeds the reference value. In the case of N.3, the 10 m height is reached at 45 s, while for the N.5 case, the 10 m height is reached at 42 s. Another clear observation that one can find in [43] is the fact that the erosion takes place during the hazard in the form of a channel excavated during the flowslide. By not taking into account the erosion rate, the model does not successfully reproduce the same pattern, as visible in simulation N.4. 

**Figure 14:**
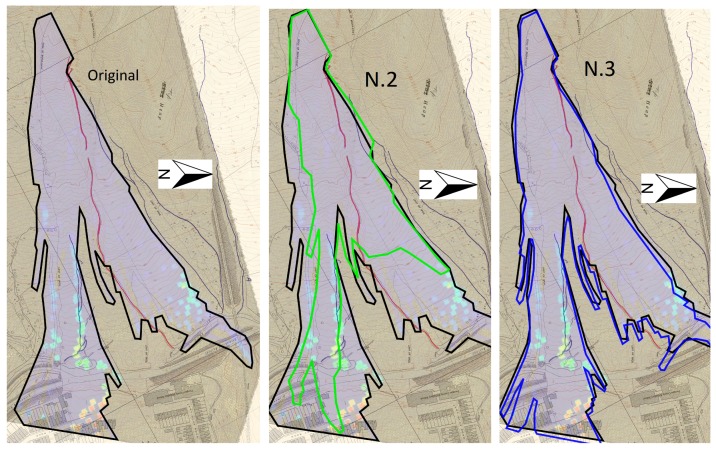
Propagation profiles: original (**left**), N.2 (**centre**) and N.3 (**right**).

**Figure 15:**
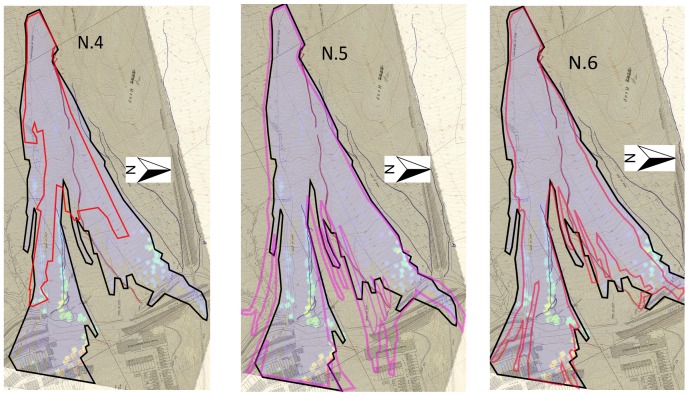
Propagation profiles: N.4 (**left**), N.5 (**centre**) and N.6 (**right**).

Moreover, it is also interesting to see how the propagation profiles change with respect to the different parameters used as in Figure 14 and Figure 15.

For simulation N.2, N.4 and N.6, it is clearly visible that the propagation profile does not reach its extension either in longitude (N.4) or width (N.2). For simulation N.3 and N.5, the profiles are much more similar to the original, especially the N.3, but they tend to exceed the propagation extension (N.5). Moreover, as comparable in Figure 5, the propagation profile is somehow homogeneous, while for both N.3 and N.5, several small branches are visible which do not replicate the real hazard or the division into two lobes as well as the parameters used for the original simulation.

## 6. Conclusions

A depth-integrated model, based on the $v-p_{w}$ Biot–Zienkiewicz formulation enhanced with a diffusion-like equation to account for the pore pressure evolution, is presented. This paper clarified the influence of the selection of the viscous model and proposed a rheological model based on Perzyna viscoplasticity. In a landslide, the pore pressure–shear stress interaction cannot take place when a pure cohesive viscous model is used. The difference between pure cohesive and frictional rheological behaviour is discussed. 

This approach, which couples solid skeleton and pore fluid, has been applied in order to simulate two case studies: the Aberfan flowslide and Gypsum tailings impoundment flowslide that both occurred in 1966. By combining the SPH technique with a set of Finite Differences, it has been possible to gain insight into the pore water pressure developed during the hazard due to changes in height, vertical consolidation and changes in total stresses.

The application of the methodology proposed and the results obtained show its suitability to be applied in studies of the propagation phase of fast landslides. The results of the Gypsum tailings impoundment flowslide agree well with the observations reported in terms of runout. Aberfan flowslide turns out to be an excellent example where the trajectory of the flowslide matches well with that which occurred, especially when the final bifurcation takes place. Moreover, the final height of the simulation also matches with the one reported in [43] and satisfactory profiles of the pore water pressure evolution varying through time are reached.

## Figures and Tables

**Table 1:** Aberfan flowslide characteristics.

Concept	Value	Meaning
Height	67 m	Height measured from the toe of the slope
Slope Terrain	12 degrees	Slope of the underlying terrain
Distance	275 m	Distance before division into two lobes
Total distance	600 m	Longest distance travelled
Distance to building	450 m	Distance before the flowslide hits the first Aberfan building
Velocity	$4.5-9\frac{m}{s}$	Estimated velocity of the flowslide

**Table 2:** Parameters used for the numerical simulation.

Parameter	Value	Meaning
$tan\phi^{\prime }$	0.726	Tangent of the friction angle
*N*	1	Perzyna model parameter
γ	0.001 s^−1^	Fluidity parameter
ρ	1740 kg·m^−3^	Material density
$e_{R}$	65 × 10^−4^ m/s	Erosion coefficient
$C_{v}$	65 × 10^−5^ m^2^/s	Consolidation coefficient
$p_{w}^{rel}$	0.8	Initial pore pressure (relative to liquefaction)
$h_{w}^{rel}$	0.4	Initial height of basal saturated layer (relative to *h*)

**Table 3:** Parameters variations for the parametric study.

Simulation	tan*ϕ*	N	Erosion (m/s)	Viscosity (s)
Original	0.726 (36°)	1	65 × 10^−4^	0.001
N.2	0.726	1	65 × 10^−4^	0.1
N.3	0.726	1	65 × 10^−4^	0.00001
N.4	0.726	1	0	0.001
N.5	0.577 (30°)	1	65 × 10^−4^	0.001
N.6	0.839 (40°)	1	65 × 10^−4^	0.001

**Table 4:** Maximum height values for the parametric study.

Max Height (m)				
	10 s	20 s	30 s	50 s
Original	5.3992	6.3663	7.6619	9.945
N.2	5.3656	5.7473	5.8304	5.6467
N.3	5.6658	6.39	7.3053	11.962
N.4	5.426	5.1076	5.3488	4.2539
N.5	5.3251	6.8389	7.6855	12.899
N.6	5.3675	6.5131	7.5588	7.8869
